# Dedifferentiated Mediastinal Liposarcoma: A Case Report

**DOI:** 10.7759/cureus.62825

**Published:** 2024-06-21

**Authors:** Elissavet Anestiadou, Anastasia Tsakona, Sokratis Tsagkaropoulos, Christoforos Foroulis, Angeliki Cheva

**Affiliations:** 1 4th Surgical Department, Papanikolaou General Hospital of Thessaloniki, Aristotle University of Thessaloniki, Thessaloniki, GRC; 2 Pathology Department, Faculty of Medicine, Aristotle University of Thessaloniki, Thessaloniki, GRC; 3 Cardiothoracic Surgery Department, Aristotle University Medical School, American Hellenic Educational Progressive Association (AHEPA) University Hospital, Thessaloniki, GRC

**Keywords:** cardiothoracic surgery, mediastinal liposarcoma, case report, mediastinum malignancy, dedifferentiated liposarcoma

## Abstract

Liposarcoma is the most common soft tissue sarcoma type in adults, originating mainly from the retroperitoneum and lower extremities. Mediastinal liposarcomas constitute an extremely rare clinical entity of mesenchymal origin. Among subtypes, dedifferentiated liposarcoma is characterized by poor survival, but little is known about its biological behavior. We present the case of a 78-year-old male patient who presented with vague symptoms, predominantly dyspnea and chest pain. Imaging revealed a large mediastinal mass and surgical resection was performed in a piecemeal manner due to the inability to achieve a microscopically negative surgical margin (R0 resection) for the residual tumor. Histological examination confirmed the diagnosis of dedifferentiated liposarcoma. The patient’s postoperative course was uneventful, with discharge from the hospital on the 10th postoperative day. However, local recurrence was detected after two months and the patient died four months after the operation. The present case report highlights the importance of radical excision for the prevention of local recurrence and the presentation of histological characteristics of this tumor. Radical surgical resection remains the fundamental treatment, while chemo and radiotherapy may have an adjuvant role. In cases of inability to obtain negative margins, surgical debulking can offer symptomatic relief.

## Introduction

Liposarcomas are the most common type of soft tissue sarcomas characterized by adipocytic differentiation and mesenchymal origin. Liposarcomas mainly arise from the soft tissues of the extremities and the retroperitoneal region [1]. On the other hand, mediastinal liposarcoma constitutes a rare entity, accounting for fewer than 1% of mediastinal tumors, and approximately 2% of liposarcomas [2]. The literature contains reports of mediastinal liposarcomas originating from all mediastinal compartments. However, posterior mediastinum is reported to be the main site or occurrence [3]. According to the World Health Organization (WHO) Classification of Soft Tissue Tumors, mediastinal liposarcomas can be classified into four basic histological types, which include the well-differentiated (WDL)/atypical lipomatous tumor (ALT), the myxoid (MDL), the pleomorphic (PLM), and the dedifferentiated mediastinal liposarcoma (DDLP) [4]. WDL/ALT represents 40-45% of liposarcomas and occurs between the fourth and fifth decades of life, presenting a better prognosis compared to DDLP. It has been proven that some cases of WDL lapse into a spindle, non-lipogenic (rarely lipogenic) sarcoma, giving rise to DDLP. In addition, WDL and DDLP types present overlapping genetic factors, with the C-Jun pathway holding a key role in the progression from WDL to DDLP [5]. Dedifferentiated and pleomorphic subtypes have a poor prognosis [6].

DDLPs constitute an entity with vague clinical and histopathological characteristics, as well as poor data regarding prognosis and outcome due to their rarity, especially when originating from the mediastinum compartments [7]. Clinical presentation is non-specific and delayed diagnosis is common due to low-rate growth, leading to symptoms due to tumor invasion and compression of adjacent organs [6]. Management strategy includes radical surgical resection, which is the main prognostic factor. Treatment outcomes are also affected by tumor size, degree of differentiation, and site of occurrence [6].

We report a case of primary mediastinal dedifferentiated liposarcoma, with infiltration of the pericardium and diaphragm in a 78-year-old patient. This case report is presented in accordance with CAse REport (CARE) guidelines [3]. In addition, the present study aims to review the clinical, histological, and therapeutic characteristics of this rare entity.

## Case presentation

A 78-year-old Caucasian male presented to the emergency department of a tertiary care academic hospital complaining of a two-month history of fatigue, cachexia, anorexia, dizziness, chest pain, and shortness of breath. No additional symptoms, such as hemoptysis, weight loss, cough, or a history of infection, were reported. Past medical history included arterial hypertension, coronary disease with percutaneous coronary intervention 18 years ago, non-insulin-dependent diabetes mellitus, and normocytic normochromic anemia. The patient was an ex-smoker, with referred tobacco cessation 25 years ago.

**Figure 1:**
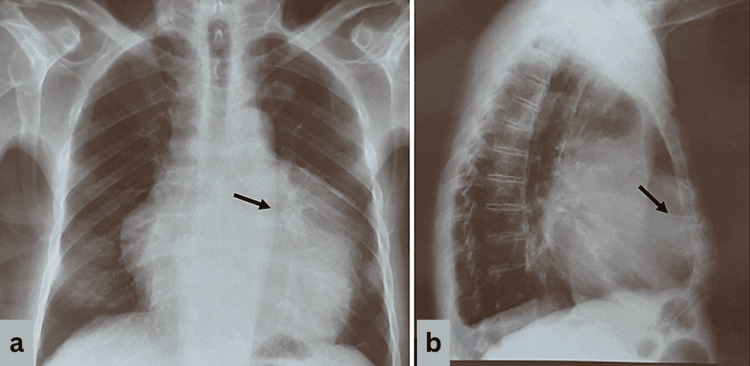
Preoperative posteroanterior upright view (a) and left lateral view (b) chest X-rays revealing a well-defined soft tissue mass occupying the left hemithorax (black arrows).

**Figure 2:**
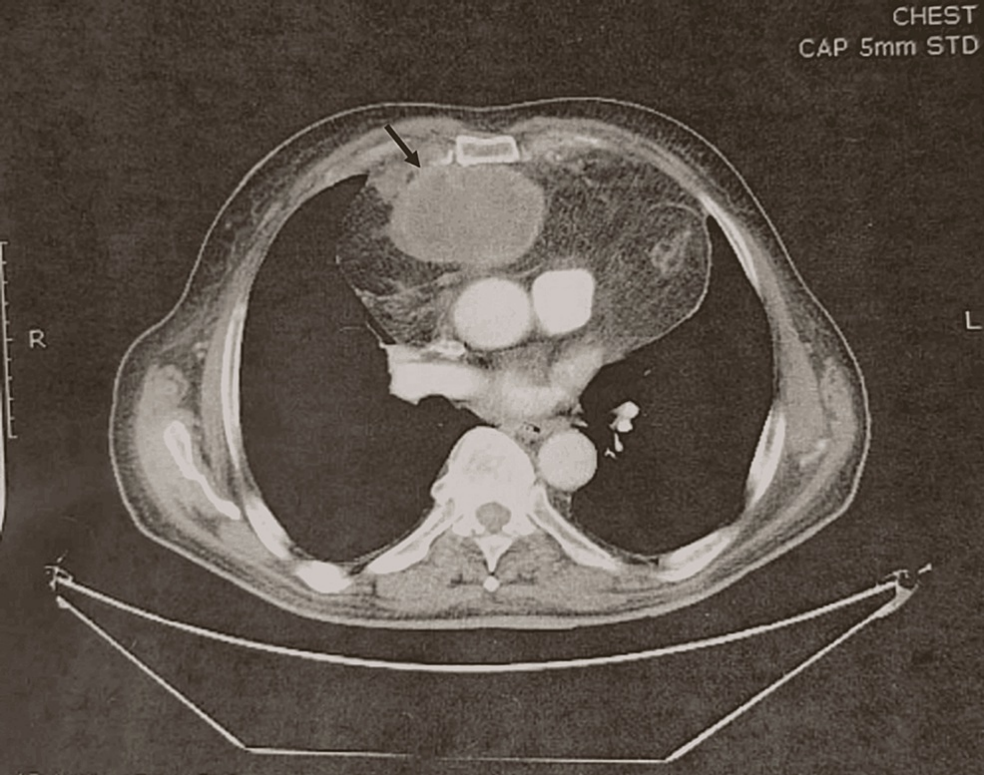
Contrast-enhanced computed tomography of the chest showing a heterogeneous cyst-fatty mass of 8.5 × 4.5 × 2 cm occupying the left hemithorax, with invasion into the pericardium and hemidiaphragm.

On physical examination, percussion of the left hemithorax revealed dullness and auscultation revealed decreased breath and heart sounds, with no additional breath sounds or murmurs noted on clinical examination. From preoperative laboratory examination, the patient’s hemoglobin was below normal limits (9.9 g/dL), while cancer antigen (CA) 15-3 levels were marginally increased (26.18 U/mL). Respiratory function tests and arterial blood gas analyses revealed normal findings. Posteroanterior (PA) upright view (Figure 1a) and left lateral view (Figure 1b) chest X-rays revealed a well-defined soft tissue mass occupying the left hemithorax, suggestive of a possible mass, while contrast-enhanced computed tomography (CT) of the chest showed a heterogeneous cyst-fatty mass measuring 8.5 × 4.5 × 2 cm occupying the left hemithorax, with invasion into the pericardium and hemidiaphragm (Figure 2), with no regional lymph node involvement. Positron emission tomography-computed tomography (PET/CT) scan was negative for metastatic disease.

**Figure 3:**
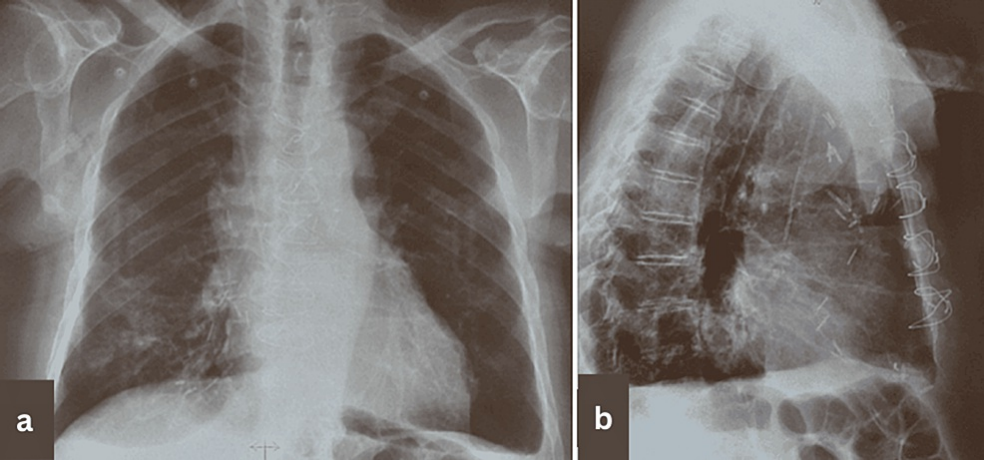
Postoperative posteroanterior upright view (a) and left lateral view (b) chest X-rays revealing clear lungs and central mediastinum.

Due to the absence of metastatic disease, surgical resection via a median sternotomy was performed under general anesthesia. During surgery, a large cystic mass measuring approximately 9 × 4.5 × 2 cm occupying the greatest part of the anterior mediastinum was found. The mass presented firm adhesions with the diaphragm and the pericardial sac, compressing the myocardium and infiltrating the pericardium at its lower part, leaving the myocardium free of invasion. Frozen biopsy failed to reveal the presence of malignancy. Piecemeal resection of the lesion was decided due to the inability to achieve microscopically margin-negative (R0) resection. Mass resection was combined with en bloc resection of the thymus and pericardial fat. The postoperative course was uncomplicated and uneventful. The postoperative chest X-rays revealed clear lungs and central mediastinum (Figures 3a, 3b). The chest tube was removed on the fifth postoperative day, with an output of 1,200 mL/24 hours. The patient was discharged from the hospital on the 10th postoperative day.

**Figure 4:**
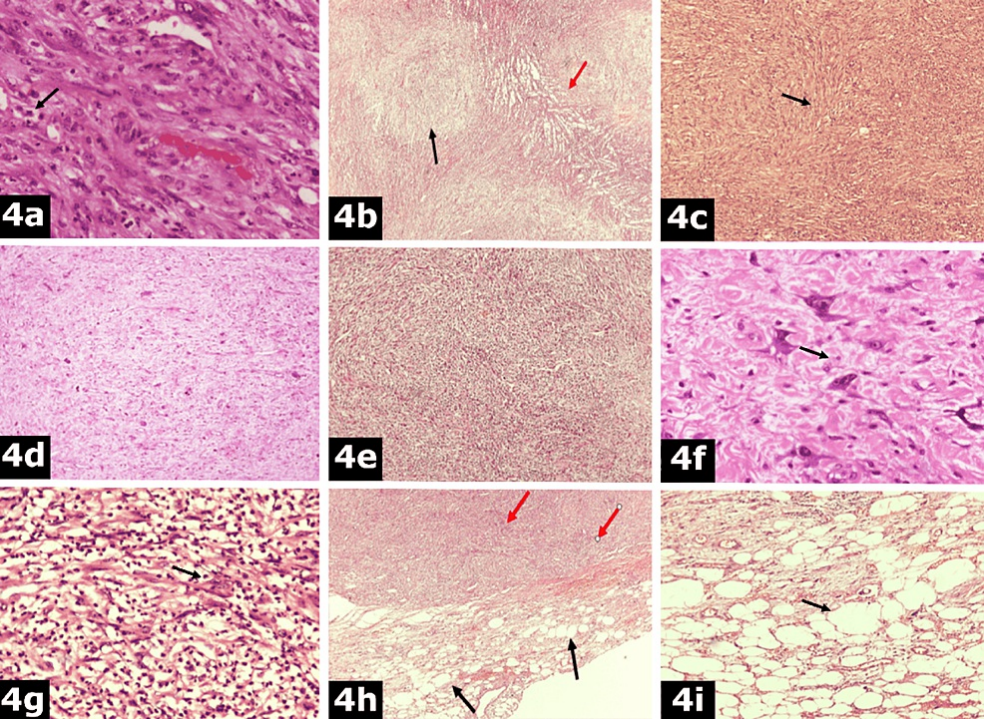
Histological examination. a: Spindle cells (black arrow) with enlarged, ovoid or round, bullous nuclei (hematoxylin and eosin stain ×400). b: Organization of neoplastic cells into fascicles (red arrow) and whorling pattern, with neural-like or meningothelial-like appearance (black arrow) (hematoxylin and eosin stain ×100). c: Neoplastic cells (black arrow) organized into a herringbone pattern (hematoxylin and eosin stain ×100). d: Areas with low cellularity (hematoxylin and eosin stain ×100). e: Areas with high cellularity (hematoxylin and eosin stain ×100). f: Multinucleated cells (black arrow) with basophilic nuclei (hematoxylin and eosin stain ×400). g: Mixed inflammatory infiltrations (black arrow) (hematoxylin and eosin stain ×200). h: Adipocytes (black arrows) and spindle cells (red arrows) (hematoxylin and eosin stain ×100). i: Different-sized adipocytes (black arrow) (hematoxylin and eosin stain ×400).

The mass was sent for histological examination. The mass measured 9 × 5 × 2 cm in diameter and the total weight was 550 g. The specimen presented an irregular contour, with one surface covered by serosa, while the opposite surface had a micronodular appearance. The cut surface showed a whitish and reddish complexion and a solid and partially cystic consistency. Microscopically, hematoxylin and eosin (H&E) stain sections revealed infiltration by stellate or spindle cells with enlarged, ovoid or round, bullous nuclei and indistinct one or more nucleoli (Figure 4a). The neoplastic cells were organized into fascicles, in a whorling pattern, with neural-like or meningothelial-like appearance (Figure 4b), herringbone pattern (Figure 4c), or irregular diffusion into a desmoplastic stroma. There were areas with high or low cellularity (Figures 4d, 4e), without the presence of adipocytes. Additionally, other cells showed an irregular outline, sharp endings, and pleomorphic, multilobulated nuclei, with one or more nucleoli. There were also multinucleated cells with basophilic nuclei (Figure 4f). Mixed inflammatory cellular infiltrations were observed, with many neutrophils (Figure 4g). Necrosis and areas with abscesses were also found in the specimen. At the periphery of the tumor, different-sized adipocytes and spindle cells with hyperchromatic nuclei were focally described into a collagenous background (Figures 4h, 4i).

**Figure 5:**
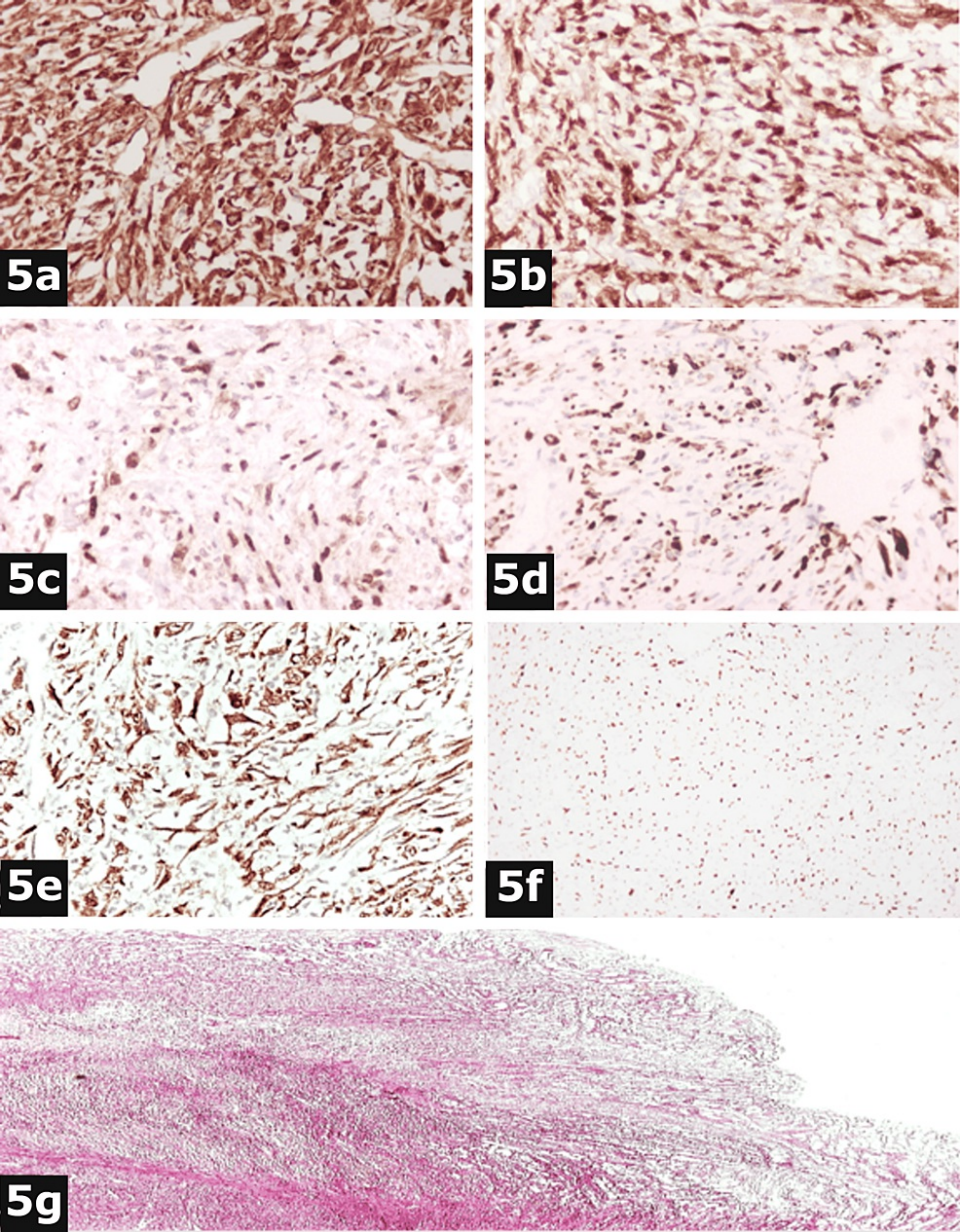
Immunohistochemistry tests. a: Vimentin stain ×400. b: CDK4 stain ×400. c: MDM2 stain ×400. d: Desmin stain ×100. e: P16 stain ×400. f: Nuclear expression in 70% of neoplastic cells (Ki67 stain ×100). g: Infiltration of pericardium by neoplastic cells (hematoxylin and eosin stain ×100).

Immunohistochemical stains revealed the following immunophenotype: (a) positive group: vimentin+ (Figure 5a), cyclin-dependent kinase-4 (CDK4+) (Figure 5b), murine double-minute type 2 (MDM2+) (Figure 5c), desmin+ (focal positivity) (Figure 5d), and p16 INK4a (p16+) (Figure 5e); and (b) negative group: anti-cytokeratin 8/18 (CK8/18-), anti-cytokeratin 5/6 (CK5/6-), anti-epithelial membrane antigen (EMA-), h-caldesmon-, calterinin-, myogenic differentiation antigen 1 (MyoD1-), anti-smooth muscle actin (SMA-), anti-S100 protein (S100-), anti-cluster of differentiation 34 (CD34-), anti-cluster of differentiation 117 (CD117-), anti-cluster of differentiation 68 (CD68-), anti-cluster of differentiation 99 (CD99-), anti-cluster of differentiation 45 (CD45-), anti-cluster of differentiation 30 (CD30-), anti-paired box protein 5 (PAX5-), and anti-Wilms’ tumor protein 1 (WT1-). Antigen Kiel 67 (Ki67) nuclear expression was presented in 70% of neoplastic cells (Figure 5f). Infiltration sites of the pericardium and pericardial fat by neoplastic cells presented morphological and immunohistochemical characteristics similar to those observed in the mediastinum (Figure 5g). The morphological and immunohistochemical findings were compatible with DDLP, with low- and high-grade components. Some areas presented the morphology of an atypical lipomatous tumor.

Postoperatively, our patient underwent two chemotherapy sessions with doxorubicin at a dose of 1.48 mg/kg due to advanced inoperable disease. Two months after surgery, a local recurrence was diagnosed in a follow-up CT scan and the patient died four months after surgical resection.

## Discussion

Mediastinal liposarcomas account for 0.1-0.75% of all mediastinal tumors. It is reported that mediastinal liposarcomas are more prevalent in males between the second and seventh decades of life than in women, demographic characteristics that also correspond to our patient [8]. All basic histologic types of liposarcoma, including WDL/ALT, MDL, PLM, and DDLP, may arise as a primary lesion in the mediastinum, with WDL/ALT being the most common subtype. Rarely, metastases of liposarcoma to the mediastinum have also been reported [9].

DDLP is typically found in the retroperitoneal cavity or proximal extremities of adults and is thought to arise from the transition of WDL/ALT to non-lipogenic sarcoma. In particular, the risk of dedifferentiation is associated with the duration of presence and site of the lesion, with the possibility being <2% in the limbs and >20% in the retroperitoneum. Regarding dedifferentiated liposarcoma origin, 90% of dedifferentiated liposarcomas develop de novo, and 10% present as recurrences [10]. Macroscopically, DDLPs are usually large-sized, multinodular, yellow, solid masses. Taking into consideration the review of Ataya et al., who reported 11 patients with giant tumors equal to or greater than 20 cm and 13 patients with tumors equal to or greater than 10 cm, it is important to mention that mediastinal liposarcomas can obtain a substantial size before becoming symptomatic [7]. Mediastinal liposarcomas usually arise from the posterior mediastinum, followed by the anterior, as in our patient, and, most rarely, the middle compartment of the mediastinum [11]. However, no significant difference has been found in the survival rates among liposarcoma locations [12].

Mediastinal liposarcomas are characterized by non-specific symptoms usually caused by compression or invasion of adjacent organs. The most commonly reported symptom is dyspnea, followed by coughing, chest discomfort and pain, hoarseness, dysphagia, and palpitations [13]. In numerous cases, mediastinal liposarcomas are incidental findings due to an asymptomatic course [7]. More rarely, Horner’s syndrome, spinal nerve paralysis, tachycardia, heart failure, and superior vena cava syndrome have been encountered and are prognostic of early mortality [14].

The diagnostic pathway includes a chest CT scan, which usually reveals a heterogeneous mass with fat densities and attenuation similar to other soft tissues. It must be highlighted that percutaneous preoperative biopsy may not be reliable in establishing a certain diagnosis due to mass heterogeneity [15]. Differential diagnosis of mediastinal liposarcoma is broad and includes all types of fat-containing mediastinal lesions, as well as other forms of small round blue cell sarcomas involving the mediastinum (Table 1) [9]. Features in favor of liposarcoma diagnosis are the location more commonly in the anterior than posterior mediastinum. They are usually large in size and can infiltrate the mediastinum. Patients are usually middle-aged and the main symptoms include dyspnea and chest pain. Finally, well-differentiated liposarcomas are characterized by large amounts of fat and soft tissue [9].

**Table 1:** Differential diagnosis of mediastinal liposarcoma.

Fat-containing mediastinal lesions
Mediastinal lipomatosis
Lipoma
Hibernoma
Lipoblastoma
Thymolipoma
Other forms of small round blue cell sarcomas
Embryonal rhabdomyosarcoma
Fibrosarcoma
Ewing’s sarcoma and variants
Lymphoma
Mesothelioma
Leiomyosarcoma
Teratoma
Neuroblastoma
Small cell carcinoma
Other lesions
Thymoma
Thymic carcinoma
Germ cell tumors
Thyroid masses
Neurogenic tumors
Pericardial cysts
Bronchogenic cysts
Esophageal tumors
Mediastinal cysts
Metastatic disease

Definite diagnosis is based on histopathological examination. The transition from a well-differentiated liposarcoma tissue to a poorly differentiated component differentiates dedifferentiated liposarcomas from other sarcomatous neoplasms [3]. According to the WHO Classification of Soft Tissue Tumors, essential diagnostic criteria include the transition of ALT to spindle, pleomorphic, non-lipogenic low or high-grade sarcomas, while desired diagnostic criteria include the expression of MDM2 or demonstration of MDM2 gene amplification [4]. Low-grade dedifferentiation is characterized by spindle cells with nuclear atypia and cellularity between WDLS and high-grade areas. High-grade dedifferentiation means either isolated, scattered adipocytes into the high-grade component or diffused, atypical, pleomorphic cells, morphologically similar to those of PLM. In cases of obscure histopathological findings, immunohistological and molecular studies, revealing overexpression of proteins or amplification of CDK4 or MDM2 genes of chromosome 12q, may support the diagnostic algorithm [8]. P16, in combination with MDM2 and CDK4, distinguishes atypical lipomatous tumors and dedifferentiated liposarcomas [16].

Although time and skill-demanding, complete surgical resection is the mainstay of treatment for mediastinal DDLPs, able to promise a low recurrence rate and cure. Invasion to adjacent structures should be managed by en bloc resection [13]. Regarding the role of adjuvant therapy, radiotherapy enhances local tumor control and decreases local recurrence rate without affecting overall survival, especially in patients with non-resectable tumors [7]. Literature reports that the response rate of mediastinal dedifferentiated liposarcoma to chemotherapy is only 25%. The combination of ifosfamide and adriamycin is the regimen of choice, while doxorubicin is also frequently administrated [14]. Despite high recurrence rates, repeat surgical resection and/or radiotherapy may be effective for local recurrence control and offer relatively long-term survival [6]. In addition, Yoshino et al. reported a case of a patient with primary dedifferentiated liposarcoma of the posterior mediastinum who had undergone positive margins and postoperative radiotherapy. The absence of recurrence after three and a half years of follow-up suggests the promising results of adjuvant radiotherapy [17]. Lately, novel agents based on MDM2 and CDK4 gene amplifications detected in dedifferentiated and well-differentiated liposarcoma are the subject of numerous ongoing clinical trials [8].

Mediastinal DDLPs present a high potential for local recurrence and distant metastasis. Chen et al., in their case series study including 23 patients with the diagnosis of primary intrathoracic liposarcoma, concluded that the dedifferentiated type is characterized by poor overall survival and recurrence-free survival period compared to the well-differentiated type, with accompanying high rates of recurrence [13]. On the contrary, Ataya et al., in their literature review, highlighted that among 13 patients with DDLP, 10 patients were recurrence-free after surgical excision, suggesting a promising prognosis [7]. Local recurrence occurs in 40% of cases. Metastases occur in 15-20% of cases and the percentage of mortality ranges between 28-30% at the five-year follow-up [6]. Literature reports for long-term prognosis after surgery are unclear [6]. In a systematic review by Kiełbowski et al. of the clinicopathological features of intrathoracic liposarcoma, the five-year survival rate was 62%, with the highest survival found in well-differentiated liposarcoma patients (80%), and the lowest in myxoid liposarcoma (31%) [18].

Postoperative follow-up should be regular and cautious to detect early local or distant recurrences. The literature contains numerous reports of mediastinal dedifferentiated liposarcoma recurrence, even after complete primary resection. Coulibaly et al. reported the case of a woman with a recurrence of mediastinal DDLP 15 months after primary resection. Despite repeat resection and adjuvant chemo and radiotherapy, the patient died due to a second local recurrence that appeared after eight years [19]. The latter case report highlights the importance of long-term strict follow-up.

This case report presents some limitations that need to be addressed. First, the patient died four months after surgery, providing only a short-term view of the disease progression and treatment efficacy. Long-term outcomes and potential recurrence patterns are not addressed. In addition, the present case involved an elderly male patient with specific comorbidities, which might not represent the broader population affected by dedifferentiated mediastinal liposarcoma. The variability in patient demographics and tumor characteristics can affect the generalizability of the findings. Overall, while the case report provides valuable insights into a rare condition, its findings should be interpreted with caution due to the inherent limitations of single case studies. Further research involving larger patient cohorts and longer follow-up periods is necessary to confirm these findings and improve the understanding of dedifferentiated mediastinal liposarcoma.

Among the key points of this case report is the fact that despite liposarcomas being the most common type of soft tissue sarcomas in adults, mediastinal liposarcomas are rare. Among the latter, DDLP presents a poor prognosis and non-specific clinical presentation, often diagnosed late due to slow growth. Radical surgical resection is crucial, while chemotherapy and radiotherapy may play adjuvant roles. DDLP is characterized by a high recurrence rate and poor overall survival, rendering regular and long-term follow-up crucial.

## Conclusions

Primary mediastinal liposarcoma is a rare mesenchymal tumor originating from adipose tissue in the mediastinum. The dedifferentiated subtype of this tumor is linked to poor prognoses, whereas the well-differentiated subtype is associated with more favorable prognoses. The preferred method of managing all cases is surgical resection with negative margins, with adjuvant radiation therapy being essential to reduce recurrence risk. The effectiveness of chemotherapy in mediastinal liposarcoma treatment is controversial and has limited benefits. The average age of mediastinal liposarcoma patients, gender prevalence, and tumor localization have yielded conflicting results, emphasizing the need for further research with larger sample sizes to yield more conclusive findings.
